# First report of the genus *Phytodietus* Gravenhorst, 1829 (Hymenoptera: Ichneumonidae: Tryphoninae) from Thailand

**DOI:** 10.3897/BDJ.4.e8027

**Published:** 2016-04-28

**Authors:** Agata Kostro-Ambroziak, Alexey Reshchikov

**Affiliations:** ‡ University of Bialystok, Institute of Biology, Department of Invertebrate Zoology, Ciołkowskiego 1J, 15-245 Białystok, Poland; § Department of Zoology, Swedish Museum of Natural History, Stockholm, Sweden

**Keywords:** Ichneumonidae, *P. longicauda*, *P. pitambari*, *P. spinipes*, parasitoid wasp, Oriental region, South East Asia

## Abstract

**Background:**

The genus *Phytodietus* Gravenhorst, 1829 is a species rich group of ichneumonid parasitoid wasps. It is represented in all zoogeographical regions, but knowledge of *Phytodietus* species in the Oriental region is patchy and restricted to some countries.

**New information:**

Here the genus *Phytodietus* is recorded from Thailand for the first time based on three species. Diagnosis and illustrations of *P. longicauda* (Uchida, 1931), *P. pitambari* Kaur et Jonathan, 1979 and *P. spinipes* (Cameron, 1905) are given. Furthermore, known distributional and biological data of the species are summarised and an identification key to the species is provided.

## Introduction

*Phytodietus* Gravenhorst, 1829 (Hymenoptera, Ichneumonidae) belonging to the subfamily Tryphoninae, tribe Phytodietini, consists of species that are koinobiont ectoparasitoids of semi-concealed larvae of several families of Lepidoptera, mainly Tortricidae and Pyralidae (Bennett 2015). The genus is distributed worldwide and currently includes 122 described species (Bennett 2015, Kasparyan and Khalaim 2013, Kostro-Ambroziak 2011a, Kostro-Ambroziak 2011b, Kostro-Ambroziak 2012, Kostro-Ambroziak and Broad 2016). To date, 22 species of *Phytodietus* have been recorded in the Oriental region: 10 species from India, 7 from Philippines, 6 from Myanmar, 5 from China, 4 from Taiwan, 3 from Indonesia and 1 from Sri Lanka (Gupta 1987, Kaur and Jonathan 1979, Yu et al. 2012). Based on distributional data, some of the *Phytodietus* species were expected to occur also in other countries of South East Asia, but no species of this genus had been recorded from Thailand so far.

Here three species of the genus *Phytodietus* are recorded as new to Thailand and an identification key to these taxa is provided.

## Materials and methods

The current study was based on material collected by the TIGER project, a collaborative effort between staff at the Queen Sirikit Botanic Garden (QSBG), the Thai Forestry Group, the Hymenoptera Institute of the University of Kentucky, and the Natural History Museum of Los Angeles County. Photographs were taken using an opto-digital microscope DSX110 in the Laboratory of Ecology and Evolutionary Biology of Insects (University of Bialystok, Poland). Morphological terminology follows Gauld et al. 1997.

## Taxon treatments

### Phytodietus longicauda

(Uchida, 1931)

#### Materials

- **Type status:** Other material. **Occurrence:** catalogNumber: T5259; recordedBy: Sirichai; individualCount: 1; sex: female; **Location:** country: Thailand; verbatimLocality: Petchaburi, Kaeng Krachan National Park, km33/helipad; verbatimElevation: 735 m; verbatimLatitude: 12°50.177’N; verbatimLongitude: 99°20.688’E; **Identification:** identifiedBy: Agata Kostro-Ambroziak; **Event:** eventDate: 25.v-1.vi.2009; **Record Level:** institutionCode: QSBG

#### Diagnosis

**Figure 1.**
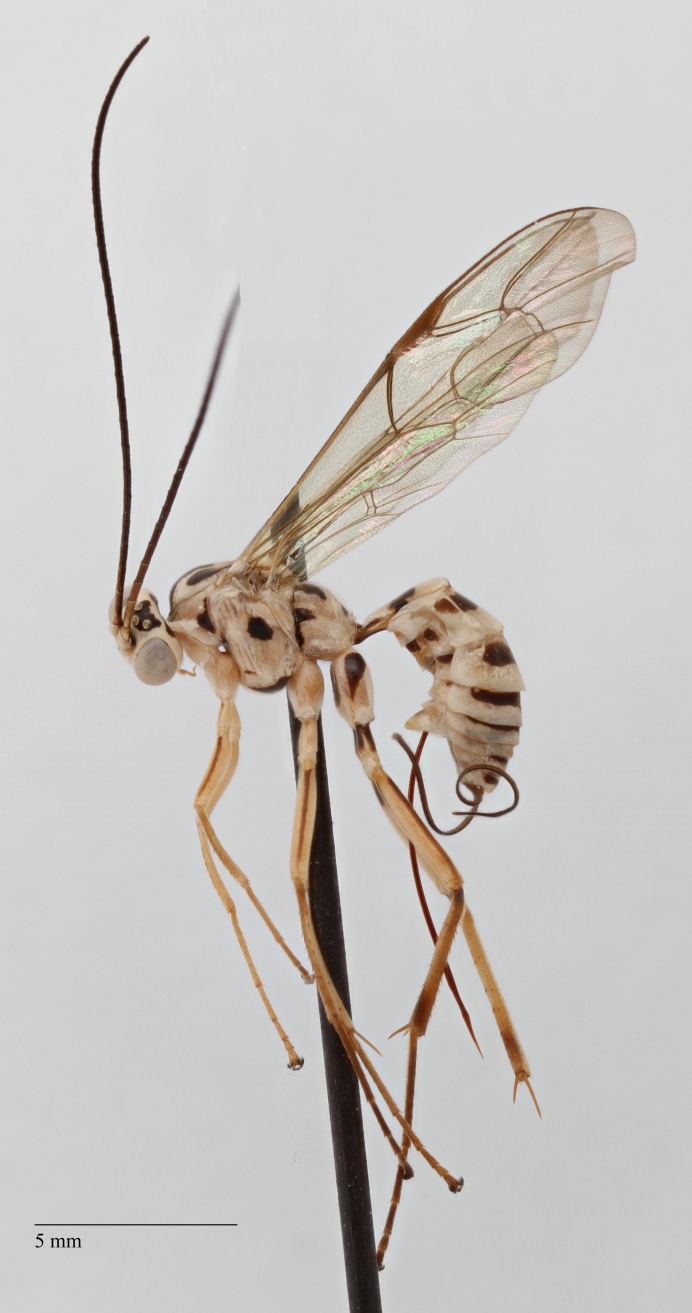
*Phytodietus longicauda*, lateral view.

*P. longicauda* (Figs 1, 2) differs from other congeners, with the exception of an undescribed species from Papua New Guinea (Kostro-Ambroziak, unpubl.), in the presence of a pleural carina (Fig. 2d). This species is distinguished from other *Phytodietus* species known from Thailand also by the following features: epomia present (Fig. 2c), distinct wrinkles on propodeum and metapleuron (Fig. 2d) and a constriction between the base and spiracles of the first metasomal segment (Fig. 3a).

#### Distribution

*P. longicauda* is one of the most widely distributed species of *Phytodietus* and has already been recorded in China, India, Japan, Myanmar, Russia and Taiwan (Yu et al. 2012).

#### Biology

*P. longicauda* probably has more than one generation per year. It has been recorded in: May in India (Kaur and Jonathan 1979) and Thailand, June in Myanmar (Kasparyan 1998), July in Amami-Oshima island within the Ryukyu Archipelago (Japan) (Momoi 1970), August in Russia (Kasparyan and Tolkanitz 1999), October in Japan (Kaur and Jonathan 1979), December in Taiwan (Cushman 1933) and Japan (Kasparyan and Tolkanitz 1999). *P. longicauda* also occurs over a wide range of altitudes and has been noted at 2000 m a.s.l. in Myanmar (Kasparyan 1998), 1700-2286 m a.s.l. in India (Kaur and Jonathan 1979), 300 m a.s.l. in Amami-Oshima island (Momoi 1970) and 735 m a.s.l. in Thailand. There are no current host records.

### Phytodietus pitambari

Kaur et Jonathan, 1979

#### Materials

- **Type status:** Other material. **Occurrence:** catalogNumber: T112; recordedBy: Areeluck Y.; individualCount: 2; sex: female; **Location:** country: Thailand; verbatimLocality: Chiang Mai, Doi Inthanon National Park, Vachiratharn Falls; verbatimElevation: 700 m; verbatimLatitude: 18°32.311’N; verbatimLongitude: 98°36.048’E; **Identification:** identifiedBy: Agata Kostro-Ambroziak; **Event:** eventDate: 2-9.viii.2006; **Record Level:** institutionCode: QSBG

#### Diagnosis

**Figure 4.**
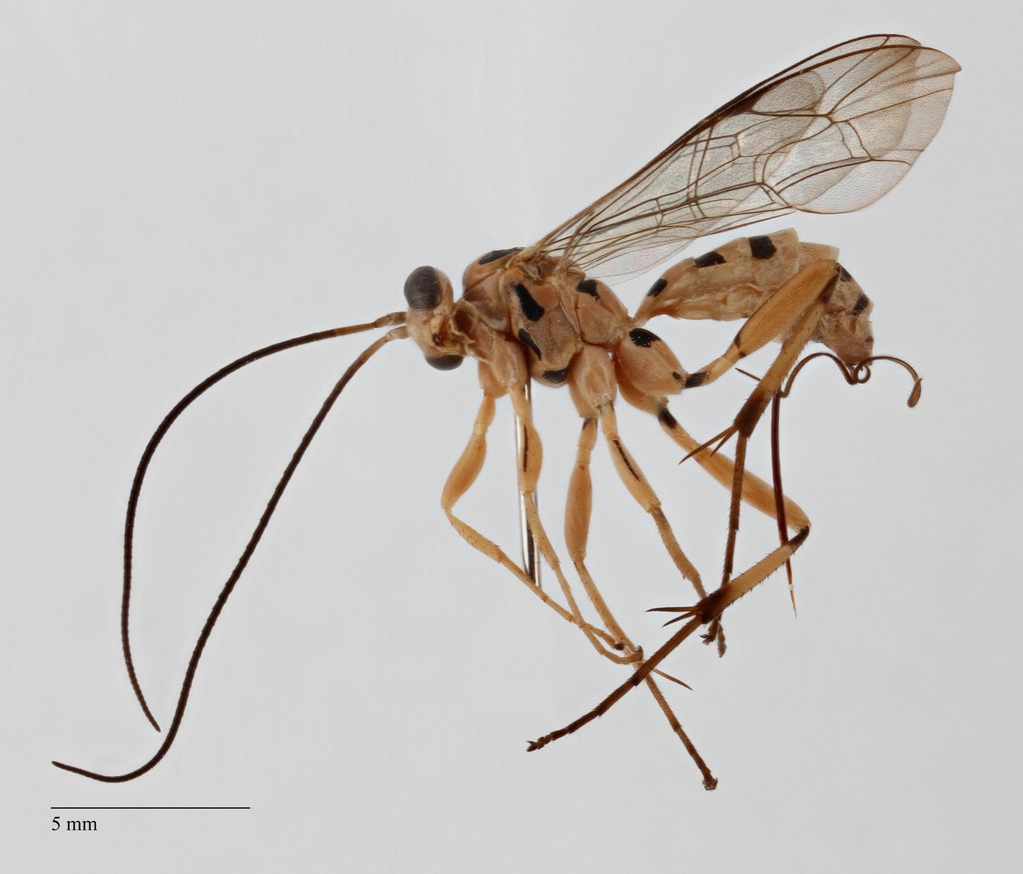
*Phytodietus pitambari*, lateral view.

*P. pitambari* (Figs 4, 5) can be easily recognized from the two congeneric species known from Thailand by the following characters: areolet of the fore wing absent (Fig. 4) and submetapleural carina not expanded anteriorly into a lobe (Fig. 6). It is distinguished from other species of *Phytodietus* lacking the areolet by having the first abscissa of *Cu* 1 shorter than *cu-a*. *P. pitambari* is similar in colour to the Oriental species *P. namkumensis* Kaur et Jonathan but differs in having the occipital carina present (absent in *P. namkumensis*) and the distance between 2*rs-m* and 2*m-cu* 1.8 times length of 2*rs-m* (3.4 for *P. namkumensis*).

#### Distribution

This species has already been recorded in India, Philippines (Jonathan 1995, Kaur and Jonathan 1979) and Japan (Shimizu and Watanabe 2015).

#### Biology

*P. pitambari* has been recorded in: April in Philippines, April and May in India (Kaur and Jonathan 1979), May, July, August in Japan (Shimizu and Watanabe 2015), and the beginning of August in Thailand suggesting that it has more than one generation per year. It has been noted at an altitude of 1228 and 610 m a.s.l. in India, and 455 m a.s.l. in Philippines (Kaur and Jonathan 1979). In Thailand *P. pitambari* was collected at 700 m a.s.l. in a mixed deciduous forest with *Dipterocarpus* sp., *Lagerstroemia* sp., *Pterocarpus macrocarpus* Kurz, *Terminalia* sp. and *Xylia xylocarpa* (Roxb.) Taub. being the dominant tree species and various grasses including i.a. *Imperata cylindrica* (L.) and *Chrysopogon zizanioides* (L.) in the shrub layer. No hosts are currently known.

### Phytodietus spinipes

(Cameron, 1905)

#### Materials

- **Type status:** Other material. **Occurrence:** catalogNumber: T6109; recordedBy: Wongchai, P.; individualCount: 1; sex: female; **Location:** country: Thailand; verbatimLocality: Chiang Mai, Doi Phahompok National Park, Doi Phaluang; verbatimElevation: 1449 m; verbatimLatitude: 20°1.06’N; verbatimLongitude: 99°9.581’E; **Identification:** identifiedBy: Agata Kostro-Ambroziak; **Event:** eventDate: 28.v.-7.vi. 2008; **Record Level:** institutionCode: QSBG
- **Type status:** Other material. **Occurrence:** catalogNumber: T2964; recordedBy: Seesom. K; individualCount: 3; sex: female; **Location:** country: Thailand; verbatimLocality: Chiang Mai, Doi Pha Hom Pok National Park, Kewlom1/montane forest; verbatimElevation: 2174 m; verbatimLatitude: 20°3.549’N; verbatimLongitude: 99°8.552’E; **Identification:** identifiedBy: Agata Kostro-Ambroziak; **Event:** eventDate: 14-21.ii.2008; **Record Level:** institutionCode: QSBG
- **Type status:** Other material. **Occurrence:** catalogNumber: T5587; recordedBy: Anuchart; individualCount: 1; sex: female; **Location:** country: Thailand; verbatimLocality: Chiang Mai, Huai Nam Dang National Park, Guest house; verbatimLatitude: 19°18.803’N; verbatimLongitude: 98°36.395’E; **Identification:** identifiedBy: Agata Kostro-Ambroziak; **Event:** eventDate: 7-14.i.2008; **Record Level:** institutionCode: QSBG
- **Type status:** Other material. **Occurrence:** catalogNumber: T5615; recordedBy: Anuchart &Thawatchai; individualCount: 1; sex: female; **Location:** country: Thailand; verbatimLocality: Chiang Mai, Huai Nam Dang National Park, Thung Buatong View Point; verbatimLatitude: 19°17.56’N; verbatimLongitude: 98°36.029’E; **Identification:** identifiedBy: Agata Kostro-Ambroziak; **Event:** eventDate: 9-10.ii.2008; **Record Level:** institutionCode: QSBG
- **Type status:** Other material. **Occurrence:** recordedBy: W. Srisuka R. Sawkord S. Pilakantha C. Sulin and T.Somboonchai; individualCount: 1; sex: female; otherCatalogNumbers: QSBG2014-67; **Location:** country: Thailand; stateProvince: Chiangmai; county: Fang; locality: Doi Pha Hom Pok National Park; verbatimLocality: Route to summit; verbatimElevation: 2036; verbatimLatitude: 20°03'01.5"N; verbatimLongitude: 99°08'38.6"E; **Identification:** identifiedBy: Alexey Reshchikov; **Event:** samplingEffort: Malaise trap; verbatimEventDate: 28.i-28.ii.2014; **Record Level:** institutionCode: QSBG
- **Type status:** Other material. **Occurrence:** recordedBy: W. Srisuka R. Sawkord T. Somboonchai and S. Suriya; individualCount: 1; sex: female; otherCatalogNumbers: QSBG2014-140; **Location:** country: Thailand; stateProvince: Chiangmai; county: Fang; locality: Doi Pha Hom Pok National Park; verbatimLocality: Route to summit; verbatimElevation: 2105 m; verbatimLatitude: 20°03'17.7"N; verbatimLongitude: 99°08'32.6"E; **Identification:** identifiedBy: Alexey Reshchikov; **Event:** samplingEffort: Malaise trap; verbatimEventDate: 1-30. iv. 2014; **Record Level:** institutionCode: QSBG

#### Diagnosis

**Figure 7.**
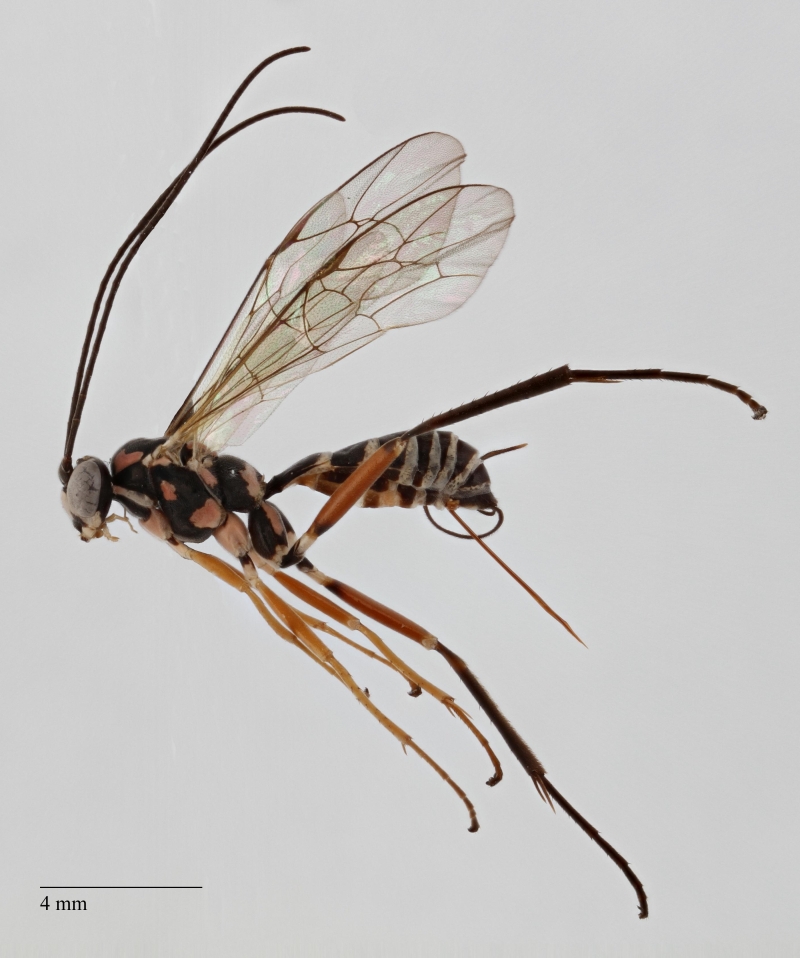
*Phytodietus spinipes*, lateral view.

*P. spinipes* (Figs 7, 8) can be distinguished from other Thai species of *Phytodietus* by having the dorsolateral margins of the first metasomal tergite sharp along the whole length (Fig. 3c, d) (mostly rounded in the other species (Fig. 3a, b)), and the body predominantly black (Fig. 7), with numerous yellow marks (predominantly yellow in the other two species (Figs 1, 4)). Among other Oriental species of *Phytodietus* which are similar in colour pattern *P. spinipes* is relatively easy to recognize by the following combination of characters: eye orbits yellow (Fig. 8a), face completely or largely yellow (sometimes very pale yellow), hind femur orange, and hind tibia and tarsus black (sometimes tibia slightly paler basally).

#### Distribution

*P. spinipes* was originally described from Sri Lanka (Cameron 1905) but it has also been recorded in China, India, Indonesia, Myanmar and Taiwan (Yu et al. 2012).

#### Biology

Data suggests that *P. spinipes* also has more than one generation per year. It has been recorded in: March in Myanmar, October in Java (Kaur and Jonathan 1979), January, February, April, May and the beginning of the June in Thailand. It has been collected at an altitude of 1000 m a.s.l. in Myanmar (Kaur and Jonathan 1979) and 1449-2174 m a.s.l. in Thailand. *P. spinipes* was collected in different types of forest in Thailand: (a) A moist evergreen montane forest with *Cinnamomum verum* J. Presl., *Prunus cerasoides* D. Don, *Schima wallichii* (DC.) Korth. and *Strychnos axillaris* Colebr. covered with mosses, ferns, lichens, orchids and other epiphytes, (b) In hill evergreen forest with *Acer oblongum* Wall. ex DC., *Anneslea fragrans* Wall., *Betula alnoides* Buch.-Ham. ex D. Don, *Litsea cubeba* (Lour.) Pers., *Magnolia hodgsonii* (Hook. f. & Thomson) H. Keng, *Pinus kesiya* Royle ex Gordon, *Quercus kingiana* Craib, *Quercus semiserrata* Roxb., (c) In a pine forest with *Pinus kesiya* Royle ex Gordon and *Pinus merkusii* Jungh. et de Vriese being the dominant tree species.

*P. spinipes* is known to be a parasitoid of *Homona coffearia* (Nietner) (Tortricidae), the tea Tortrix in Sri Lanka, Taiwan (Gupta 1987) and India (Muraleedharan and Selvasundaram 1991). In Java this species has been reared from *Homona* sp. (Kaur and Jonathan 1979).

## Identification Keys

### Key to the species of *Phytodietus* Gravenhorst, 1829 from Thailand

**Table:** 

1	Areolet of fore wing absent, submetapleural carina not expanded anteriorly into a lobe	*P. pitambari* Kaur et Jonathan, 1979
–	Areolet of fore wing present, submetapleural carina expanded anteriorly into a lobe	2
2	Pleural carina and epomia absent, body in general black with yellow marks	*P. spinipes* (Cameron, 1905)
–	Pleural carina and epomia present, body in general yellow with black marks	*P. longicauda* (Uchida, 1931)

## Supplementary Material

XML Treatment for Phytodietus longicauda

XML Treatment for Phytodietus pitambari

XML Treatment for Phytodietus spinipes

## Figures and Tables

**Figure 2a.**
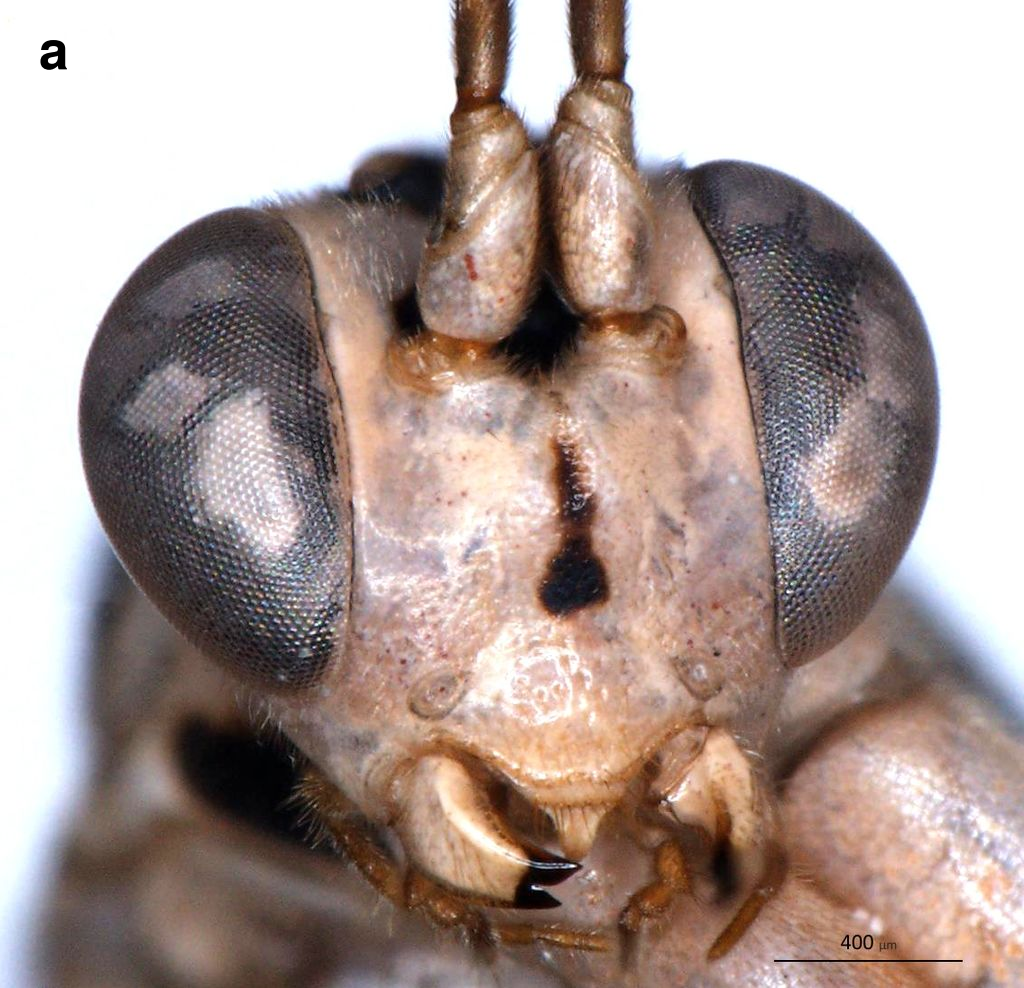
head, facial view,

**Figure 2b.**
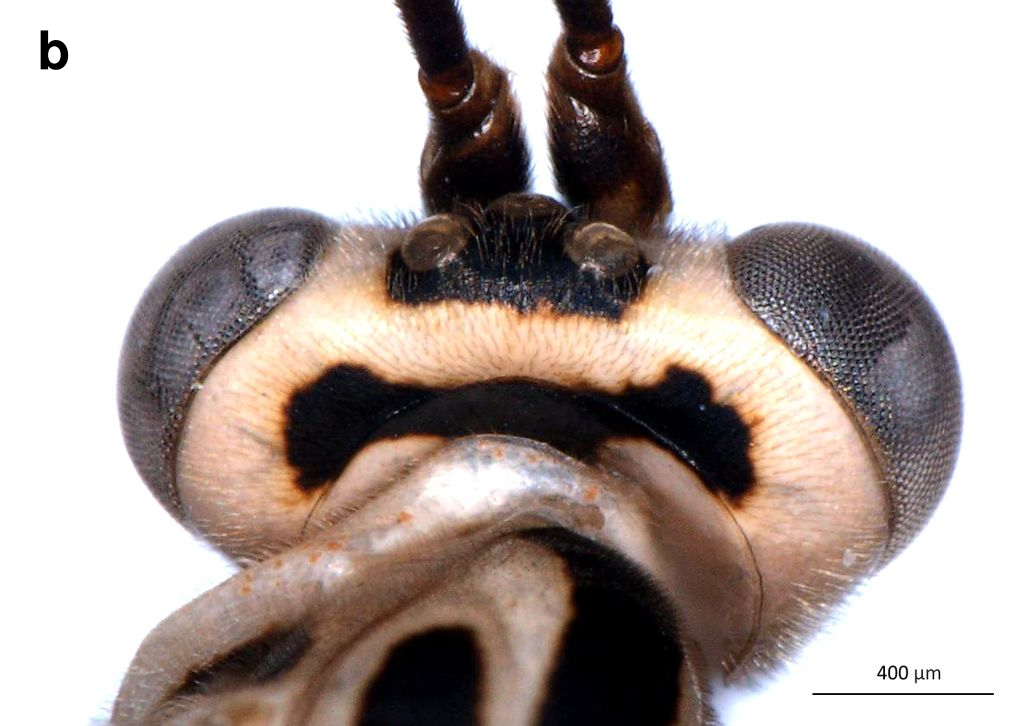
head, dorsoposterior view,

**Figure 2c.**
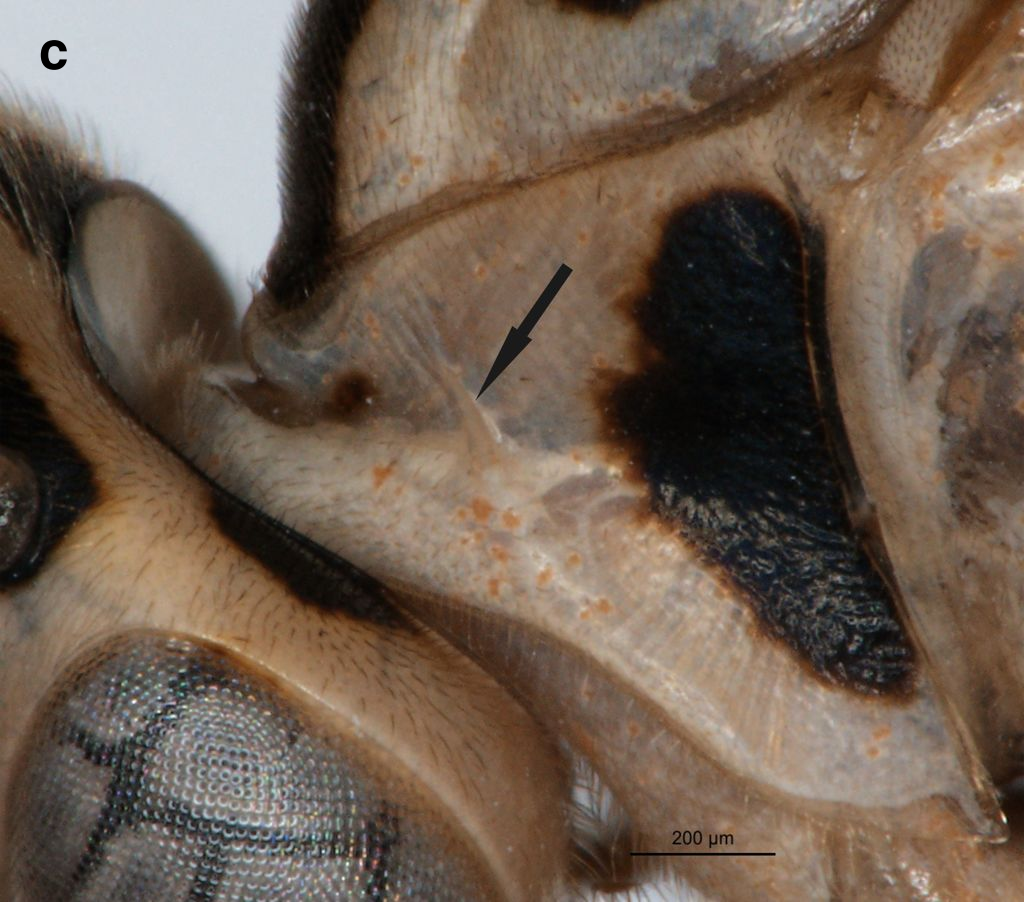
pronotum, arrow points to epomia,

**Figure 2d.**
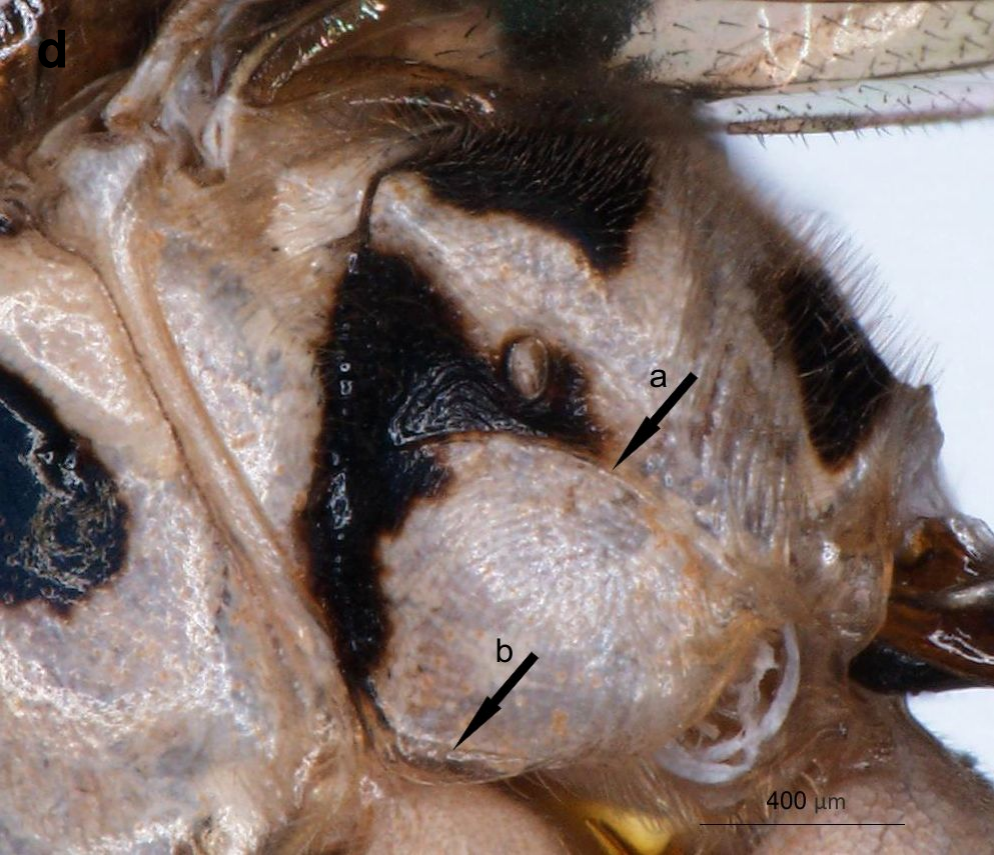
metapleuron and lateral part of propodeum, arrows point to: a - pleural carina, b - submetapleural carina expanded anteriorly into a lobe.

**Figure 3a.**
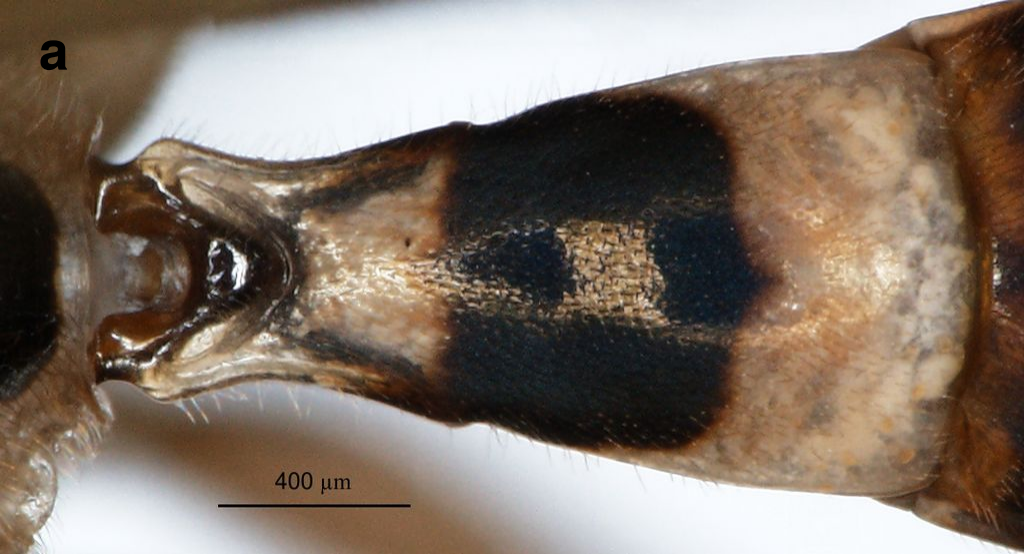
*P. longicauda*,

**Figure 3b.**
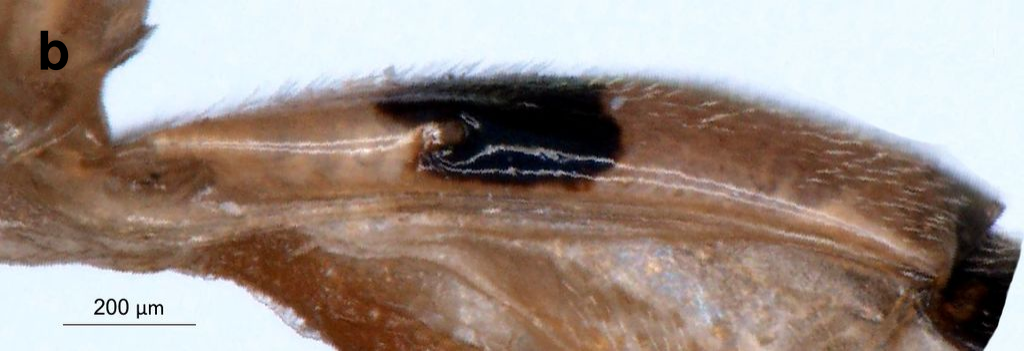
*P. pitambari*,

**Figure 3c.**
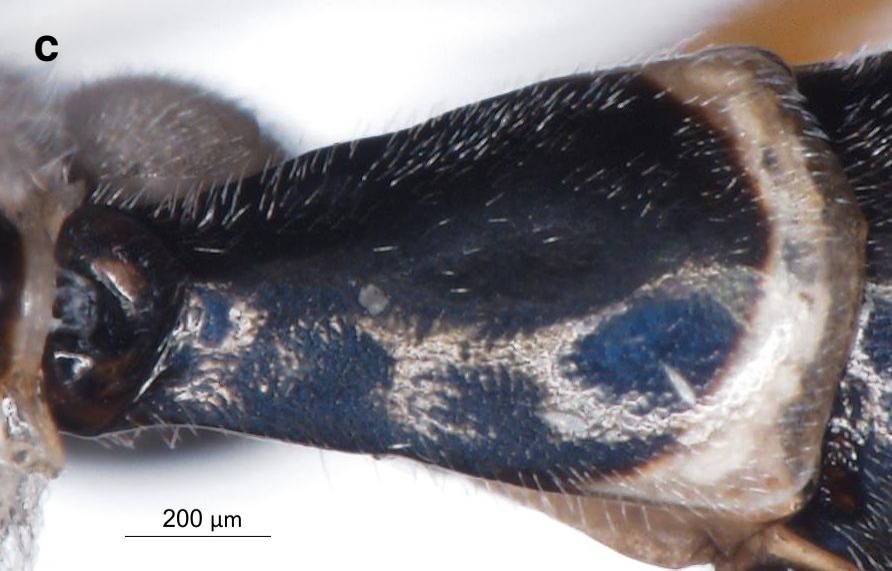
*P. spinipes*,

**Figure 3d.**
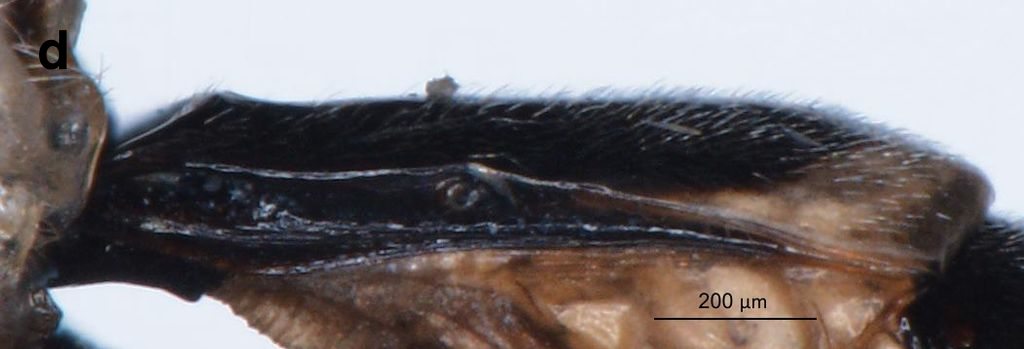
*P. spinipes*.

**Figure 5a.**
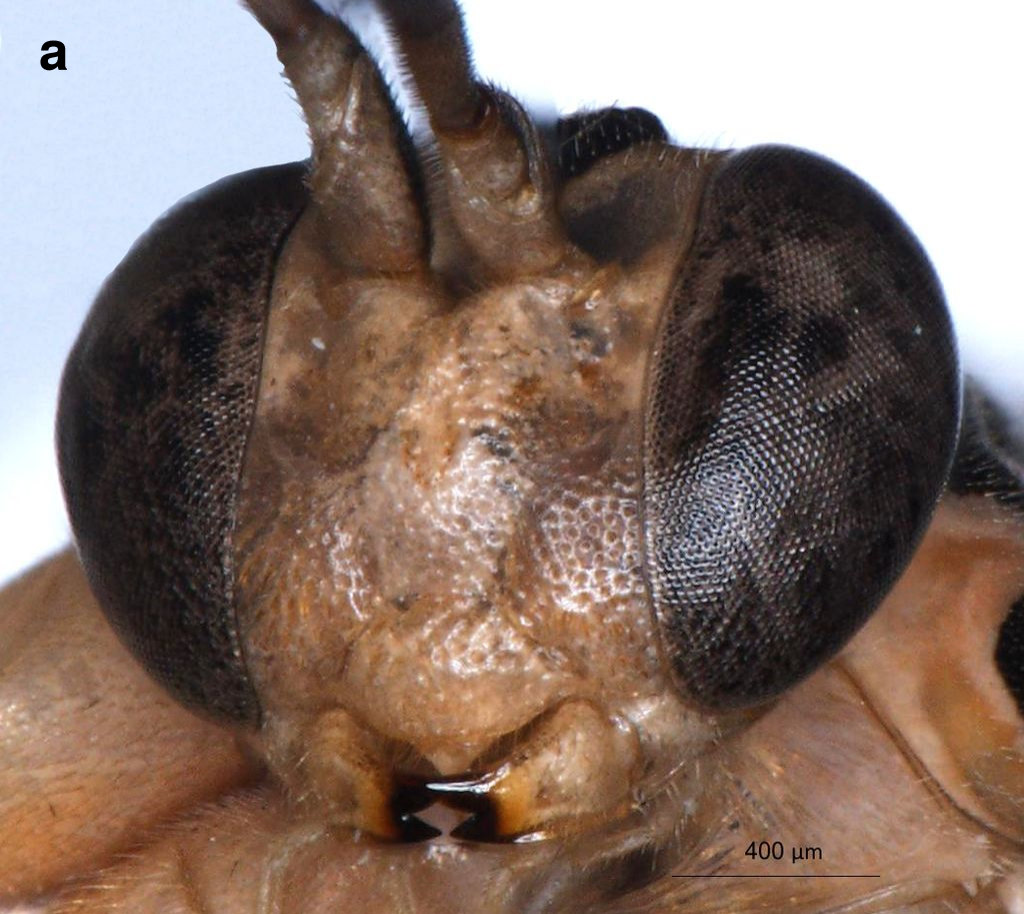
head, facial view,

**Figure 5b.**
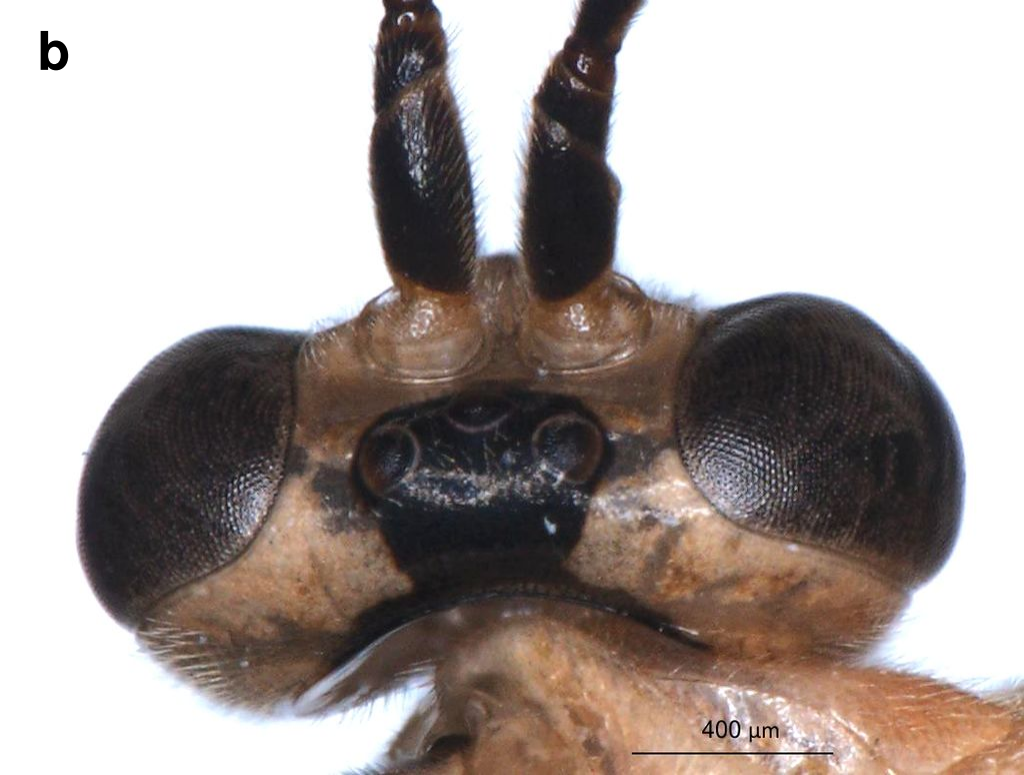
head, dorsoposterior view.

**Figure 6a.**
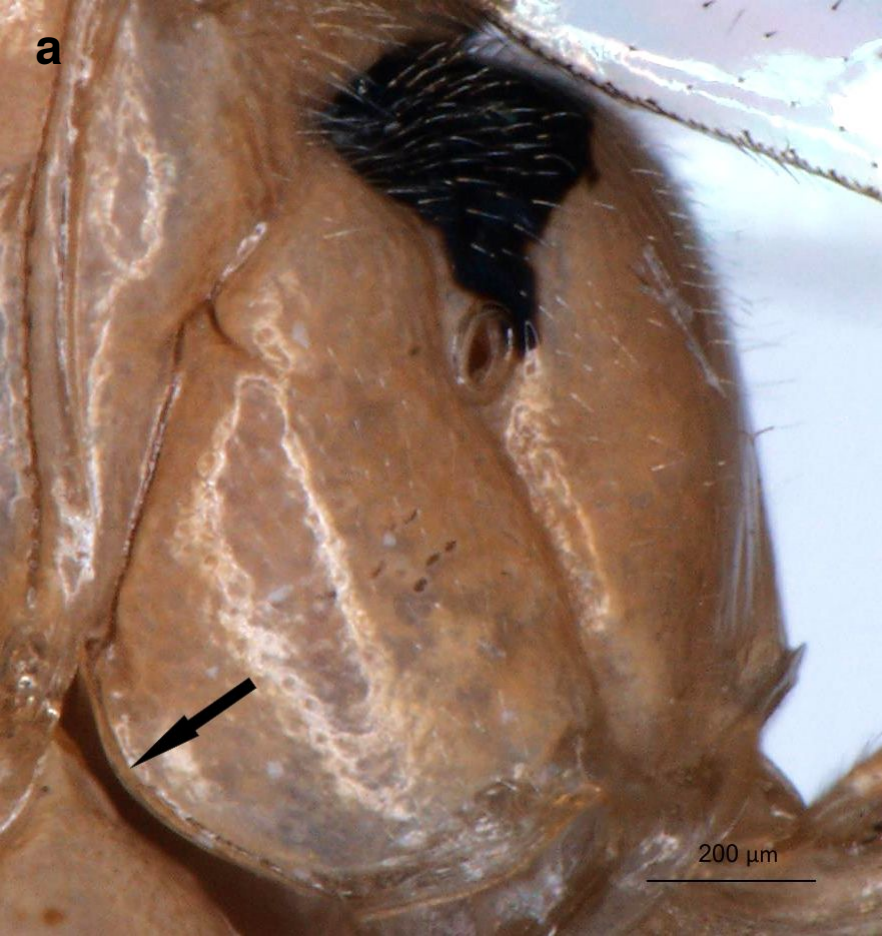
*P. pitambari*, arrow points to submetapleural carina not expanded into a lobe,

**Figure 6b.**
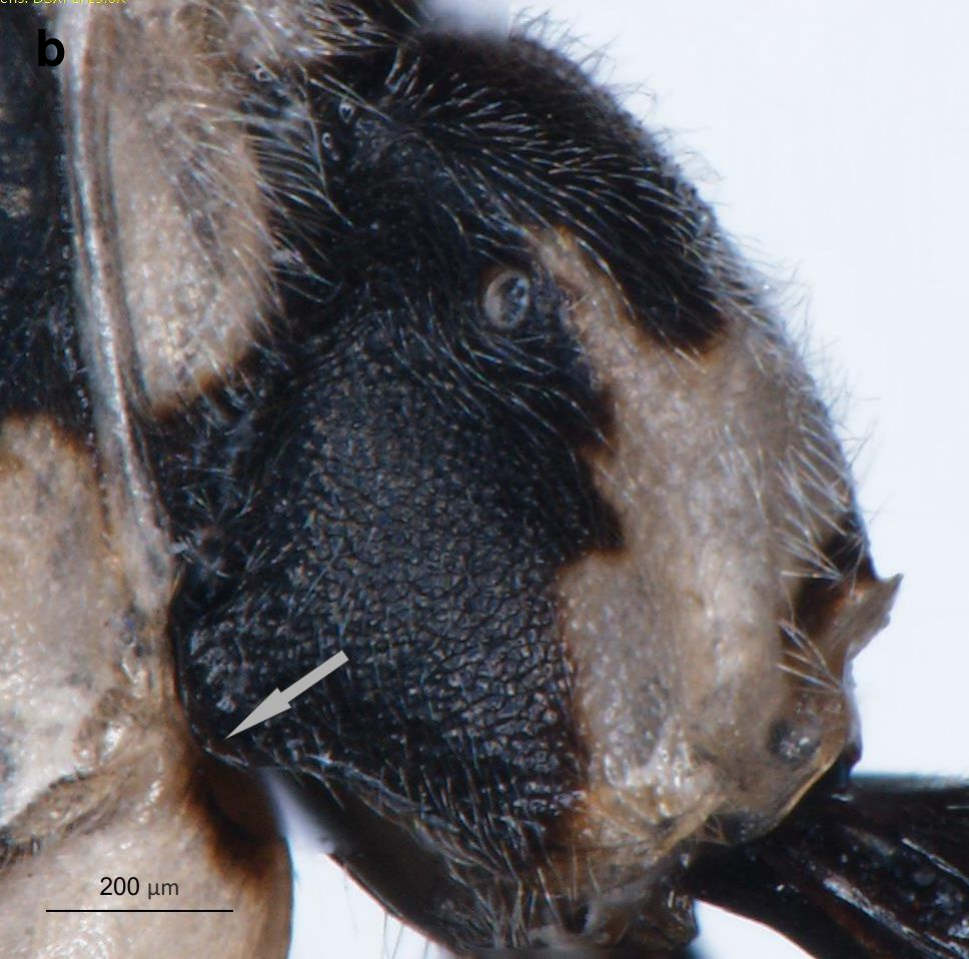
*P. spinipes*, arrow points to submetapleural carina expanded into a lobe.

**Figure 8a.**
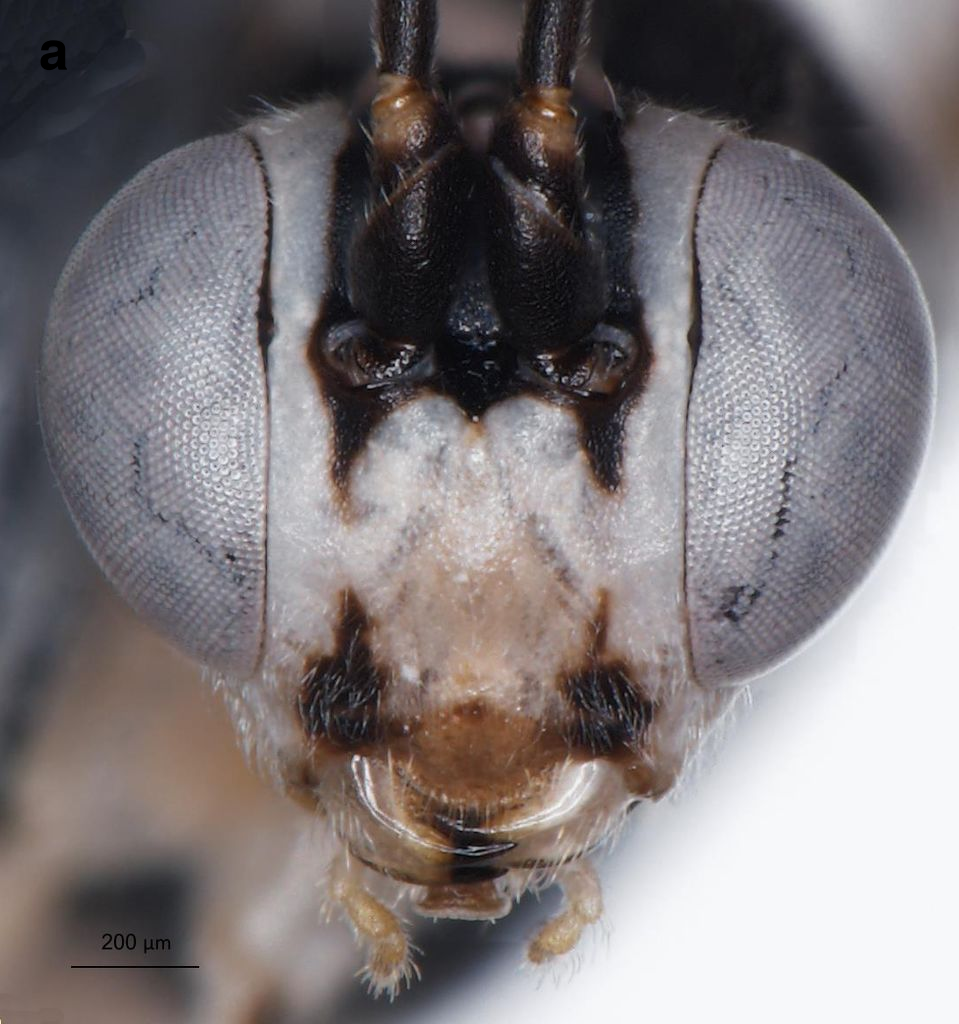
head, facial view,

**Figure 8b.**
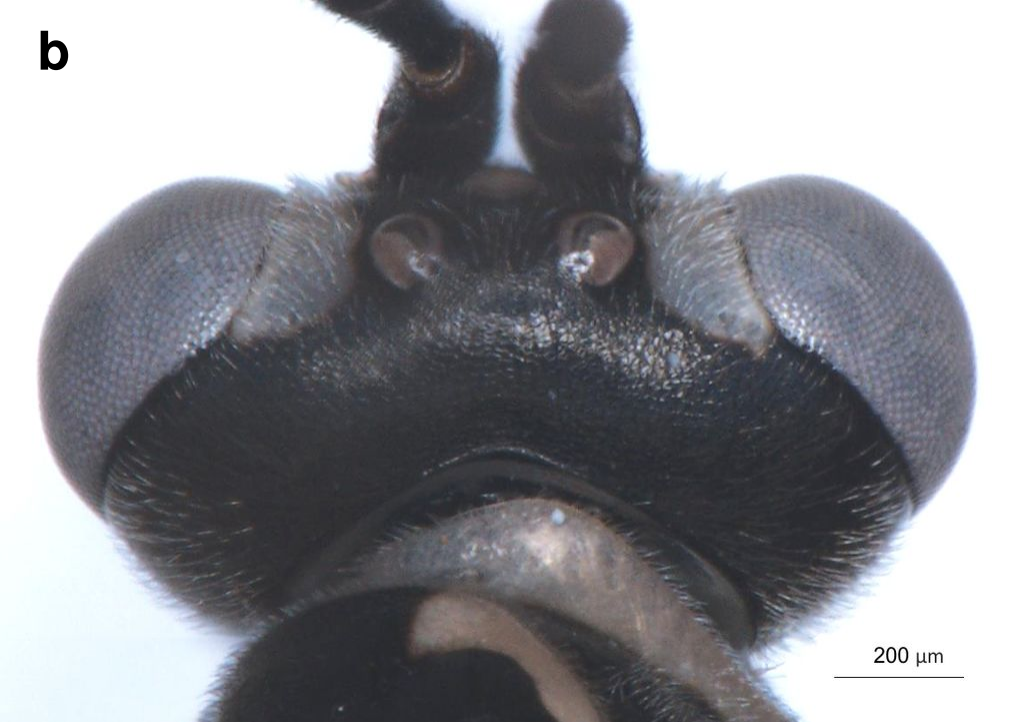
head, dorsoposterior view.

## References

[B3072012] Bennett A. M. R. (2015). Revision of the World Genera of Tryphoninae (Hymenoptera: Ichneumonidae).. Memoirs of the American Entomological Institute.

[B3072105] Cameron P. (1905). On the phytophagous and parasitic Hymenoptera collected by Mr. E. Green in Ceylon.. Spolia Zeylanica.

[B3072115] Cushman R. A. (1933). H. Sauter’s Formosa-collection: Subfamily Ichnuemoninae (Pimplinae of Ashmead).. Insecta Matsumurana.

[B2997500] Gauld I., Wahl D., Bradshaw K., Hanson P., Ward S. (1997). The Ichneumonidae of Costa Rica, 2. Introduction and keys to species of the smaller subfamilies, Anomaloninae, Ctenopelmatinae, Dipazontinae, Lycorininae, Phrudinae, Tryphoninae (excluding *Netelia*) and Xoridinae, with an appendix on the Rhyssinae.. Memoirs of the American Entomological Institute.

[B1432745] Gupta V. K. (1987). The Icheumonidae of the Indo-Australian area (Hymenoptera). Memoirs of the American Entomological Institute.

[B2997511] Jonathan J. K., Ghosh A. K. (1995). Hymenoptera: Ichneumonidae. Fauna of western Himalaya. Part 1: Uttar Pradesh..

[B1433117] Kasparyan D. R. (1998). New species of ichneumonid wasp (Hymenoptera, Ichneumonidae) collected by R. Malaise in Burma. Entomologicheskoye Obozreniye.

[B3072022] Kasparyan D. R., Khalaim A. I. (2013). A new species of the genus *Phytodietus* Gravenhorst, 1829 (Hymenoptera: Ichneumonidae: Tryphoninae) from Mexico.. Proceedings of the Zoological Institute RAS.

[B3072034] Kasparyan D. R., Tolkanitz V. I. (1999). Ichneumonidae. Subfamily Tryphoninae: tribes Sphinctini, Phytodietini, Oedemopsini, Tryphonini (Addendum), Idiogrammatini. Subfamilies Eucerotinae, Adelognathinae (Addendum), Townesioninae. Fauna of Russia and neighbouring countries. InsectaHymenoptera, New Series..

[B1433127] Kaur R., Jonathan J. K. (1979). Ichneumonologia Orientalis, Part VIII. The tribe Phytodietini from India (Hymenoptera: Ichneumonidae).. Oriental Insects Monograph.

[B3072046] Kostro-Ambroziak A. (2011). Phytodietus (Weisia) pearlus sp. nov. from South Africa (Hymenoptera: Ichneumonidae).. Annales Zoologici.

[B3072056] Kostro-Ambroziak A. (2011). A new species of *Phytodietus* Gravenhorst, 1829 (Hymenoptera: Ichneumonidae: Tryphoninae) from North Africa.. Entomological News.

[B3072067] Kostro-Ambroziak A. (2012). Taxonomic study of the genus *Phytodietu* s Gravenhorst, 1829 (Hymenoptera: Ichneumonidae) from Australia, with description of a new species.. Deutsche Entomologische Zeitschrift.

[B3072077] Kostro-Ambroziak A., Broad G. (2016). Genus *Phytodietus* Gravenhorst, 1829 new to South America, with description of a new species (Hymenoptera, Ichneumonidae, Tryphoninae).. Annales Zoologici.

[B3072087] Momoi S. (1970). Ichneumonidae (Hymenoptera) of the Ryukyu Archipelago.. Pacific Insects.

[B2997490] Muraleedharan N., Selvasundaram R. (1991). Bioecology of *Phytodietus
spinipes* (Cameron) a parasitoid of *Homona
coffearia* Nietner, the tea Tortrix. Journal of Plantation Crops.

[B2997479] Shimizu S., Watanabe K. (2015). The Subgenus *Weisia* Schmiedeknecht, 1907, of the Genus *Phytodietus* Gravenhorst, 1829 (Hymenoptera: Ichneumonidae: Tryphoninae), New to Japan and Eastern Palearctic Region.. Japanese Journal of Systematic Entomology.

[B1433138] Yu D. S., Achterberg K., Horstmann K. (2012). World Ichneumonoidea 2011. Taxapad 2012..

